# Management of insertional Achilles tendinopathy through a Cincinnati incision

**DOI:** 10.1186/1471-2474-8-82

**Published:** 2007-08-15

**Authors:** Michael R Carmont, Nicola Maffulli

**Affiliations:** 1 Department of Trauma and Orthopaedics, University Hospital of North Staffordshire, Keele University School of Medicine, Stoke on Trent, ST7 4QG UK

## Abstract

**Background:**

About 10% of patients not responding to 3–6 months of conservative management for insertional Achilles tendinopathy undergo surgery. Traditionally, surgery of the Achilles tendon is performed through longitudinal extensile incisions. Such surgery is prone to the complications of wound healing, wound breakdown and iatrogenic nerve injury.

**Methods:**

We describe our current method of exposure of the Achilles tendon insertion and debridement of the peritendinous and tendon tissue with osteotomy of the calcaneum through a transverse skin incision at the level of the Achilles insertion.

**Results:**

This method has been used since 2002 on over 40 patients for exposure of the Achilles tendon insertion and the distal Achilles tendon.

**Conclusion:**

The Cincinnati incision allows adequate exposure, has minimal risk of symptomatic iatrogenic nerve injury, and has minimal problems related to the scar.

## Background

Achilles tendon disorders have been divided into insertional and non-insertional [1]. The distal portion of the Achilles is affected in 24% of patients with Achilles tendinopathy [2], but the actual incidence of insertional Achilles tendinopathy is unknown. Histology of recalcitrant calcific insertional tendinopathy has shown fibrocartilagenous or calcifying metaplasia, with no evidence of inflammation at the tendon insertion [3]. Conservative treatments for Achilles tendinopathy include rest, ice, non-steroidal anti-inflammatory drugs, careful footwear selection and activity avoidance, leading to the resolution of symptoms in most patients. Eccentric exercises of the gastro-soleus complex, although beneficial for midportion tendinopathy, are not as effective for insertional tendinopathy, helping in only 32% of patients [4]. Extra-corporeal sock wave therapy is effective [5], but this modality may not be readily available. Surgery is the mainstay of management for the 10% of patients not responding to 3–6 months of conservative management [6].

Traditionally, surgery of the Achilles tendon is performed through longitudinal extensile incisions. Achilles tendon surgery is prone to the complications of wound healing, wound breakdown and iatrogenic nerve injury [7].

We describe our current method, used since 2002 on over 40 patients, of exposure of the Achilles tendon insertion and the distal Achilles tendon. Using this approach, a wide exposure of the insertion of the Achilles tendon is possible, and, though it, debridement of the peritendinous and tendon tissue, with, if necessary, superficial and deep Achilles bursectomy. In addition, the transverse skin incision at the level of the Achilles insertion also allows osteotomy of the postero-superior corner of the calcaneum.

## Methods & Results

**Figure 1:**
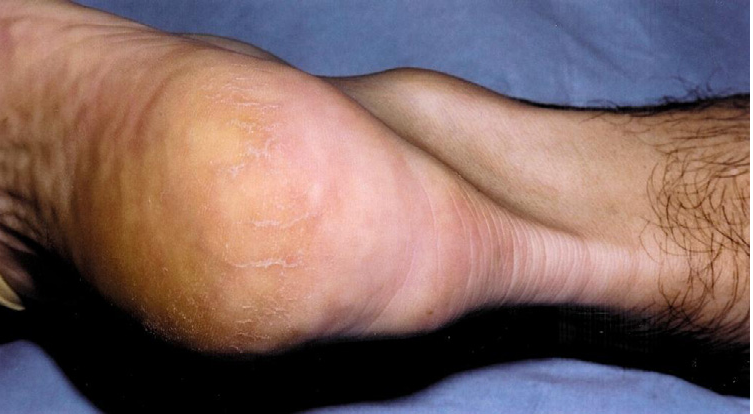
A 5 cm semi-circumferential incision is made at the level of the Achilles tendon insertion over the tendinopathic area.

**Figure 2:**
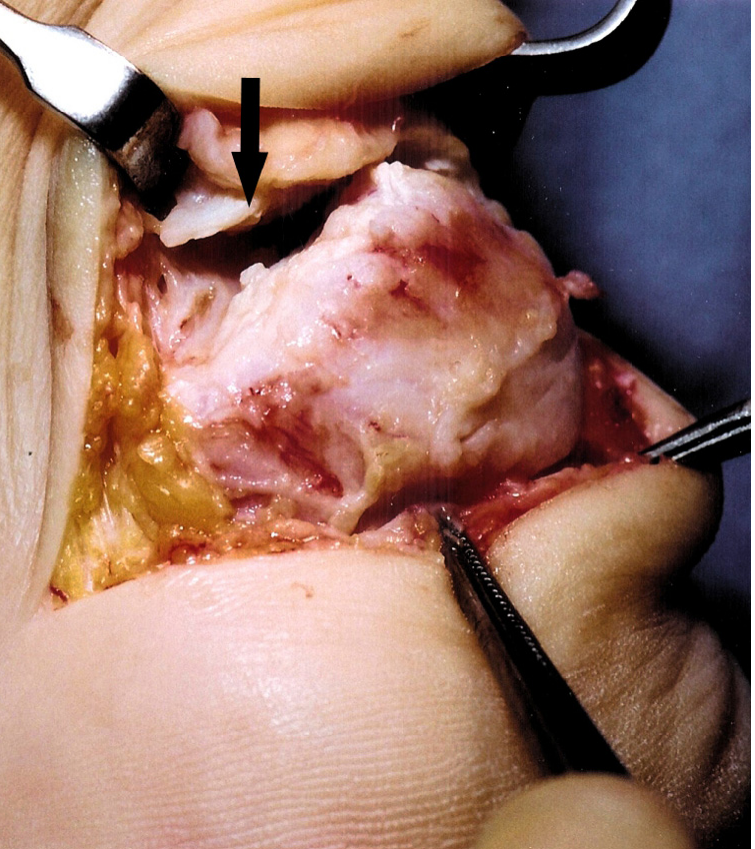
After dissection and debridement, the tendinopathic tendon insertion (solid arrow) is detached from the calcaneum.

The patient is positioned prone, a calf tourniquet is used, and prophylactic antibiotics are administered. The skin is prepared with chlorhexidine and steridrapes. Landmarks include the Achilles tendon and the tuberosity of the calcaneum. A 5 to 7 cm semicircular skin incision is made over the area of insertional tendinopathy, which is typically erythematous and swollen, with a prominent calcaneal tuberosity (Figure 1). After accurate haemostasis, the tendinopathic area of insertion is identified and detached from the calcaneum (Figure 2).

**Figure 3:**
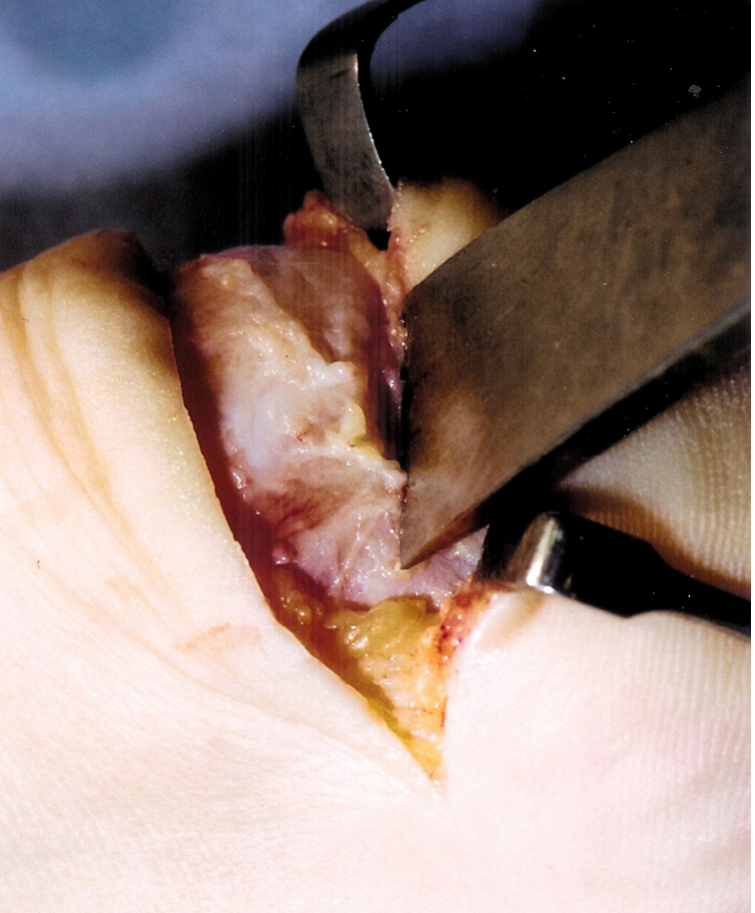
The prominent calcaneal tuberosity is detached from the calcaneum using an osteotome.

**Figure 4:**
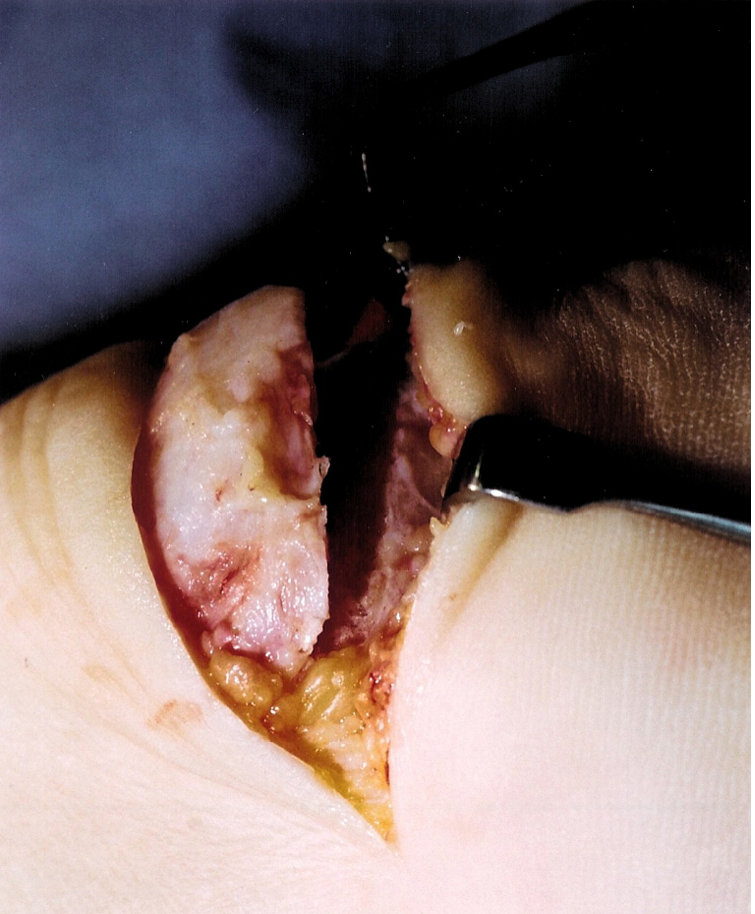
The underlying cancellous bone is now exposed.

**Figure 5:**
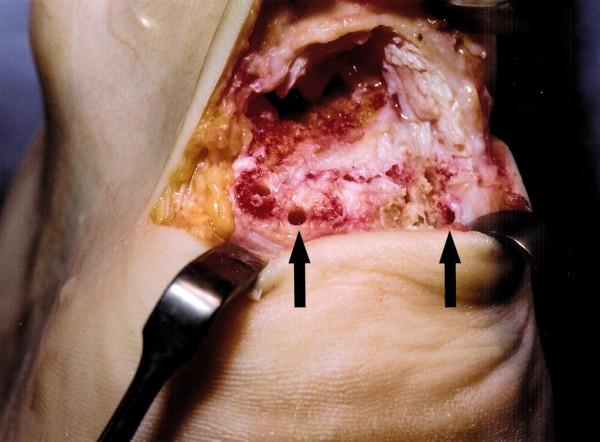
A 2.5 mm drill is used to pass holes (solid arrows) through the calcaneum to allow suture fixation to the calcaneum. The calcaneum is devoid of soft tissue as all tendinopathic tissue has been debrided.

**Figure 6:**
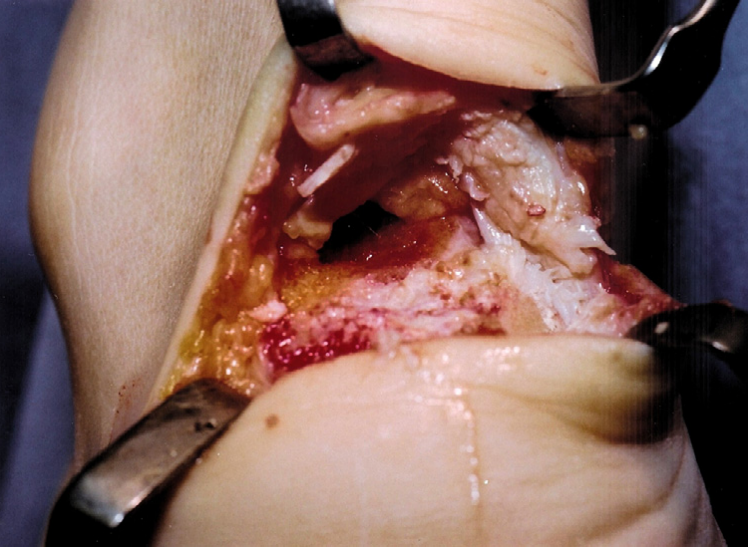
The exposed surface of the calcaneum is smoothed using a wrasp.

**Figure 7:**
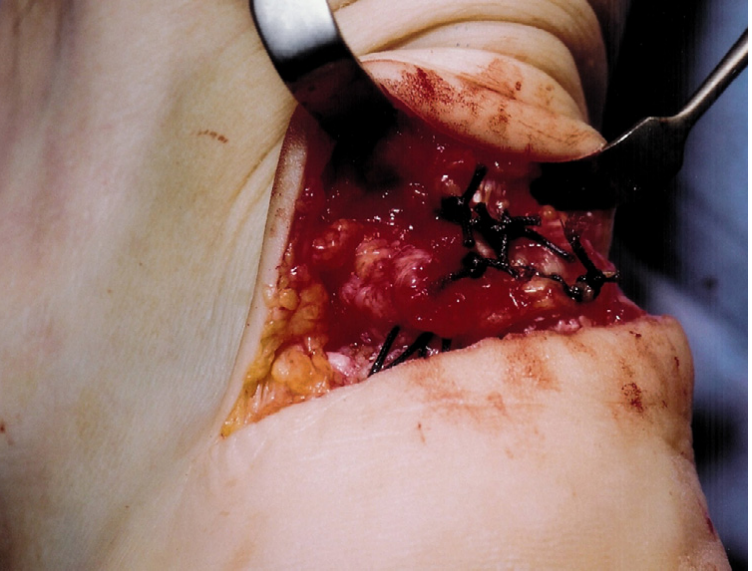
Strong absorbable suture material is used to reinsert the Achilles tendon to the calcaneum.

The tendinopathic tissue is then debrided to normal tendon tissue. The prominent tuberosity is detached using a 1 cm osteotome (Figures 3 and 4). A 2.5 mm drill is then used to perforate the calcaneum to allow the passage of sutures (Figure 5). The surface of the calcaneum is smoothed to minimise sharp prominences, and roughened to expose the cancellous bone using a rasp (Figure 6). Number 1 Vicryl (Ethicon, Edinburgh), a strong absorbable suture, is then used to reattach the tendon (Figure 7). Mitek anchors (DePuy-Mitek) may be used if the tendon end cannot be secured through the drill holes.

**Figure 8:**
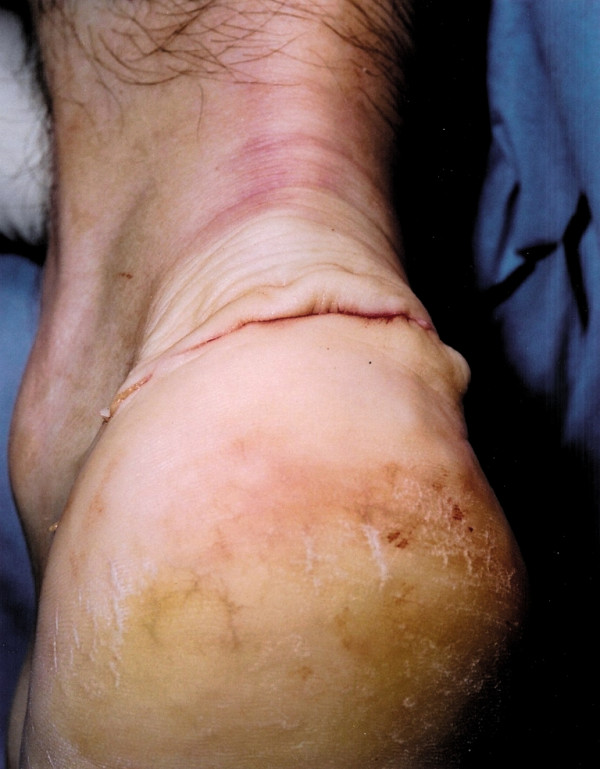
The wound is closed using subcuticular sutures.

The skin edges are opposed using 3.0 Biosyn (Tyco Health Care, Norwalk, CT) (Figure 8), a Mepore (Molnlycke Health Care, Gothenburg, Sweden) dressing is applied, and the ankle and hindfoot are immobilised in a removable below knee weight bearing cast in a plantigrade position. If the tendon was not detached from the calcaneum, the cast is removed at two weeks. Otherwise, at two weeks the cast is bivalved, and a front slab with Velcro straps is applied. The patient is asked to perform plantar flexion, eversion and inversion exercises with proprioception training. After 6 weeks, the patient mobilises free from cast and commences physiotherapy to regain ankle and subtalar movements.

## Discussion

Surgical management of insertional tendinopathy of the Achilles tendon should allow debridement of calcific areas of the tendon and permit decompression of the retrocalcaneal bursa and the superior calcaneal tuberosity [8]. This bony prominence on the superior aspect of the calcaneum, is associated with insertional Achilles tendinopathy [9]. Detachment and reconstruction of the Achilles tendon insertion including a V-Y lengthening of the proximal aponeurosis through a J shaped incision had 9% wound dehiscence, 4.6% infection rates, and 3% sural neuritis. Seventy four percent and 92% satisfaction rates were achieved in detached and non-detached tendon insertions respectively [10]. Complete excision of calcific deposits is recommended. This may require detaching the tendon from the calcaneum, and reattachment using suture anchors [3]. Complete detachment and reconstruction of the Achilles tendon does not decrease the working capacity of the gastrocsoleus muscle [11].

After the procedure, early mobilisation is safe in selected patients when less than 50% of the tendon has been resected [12], although using our technique we have had no patients who suffered detachment of the re-inserted Achilles tendon.

Previously described incisions include medial and J shaped incisions [13-15], lateral incision [7,8,16], and a combination of both medial and lateral incisions [1,17]. Recently, a central tendon splitting approach has been described allowing access to the more commonly affected central portion of the tendon with the peripheral fibres being spared [18,19]. Endoscopic procedures on the retrocalcaneal space have been described, but they cannot address the intratendinous pathology [20].

Iatrogenic sural nerve injury is a risk with all surgery to the hindfoot. The sural nerve lies 18.8 mm from the lateral border of the Achilles tendon at its insertion, then passes proximally towards the midline so that it passes the lateral border of the tendon 9.8 cm from the calcaneum [21,22]. Iatrogenic nerve damage is relatively frequent, and surgical incisions may be made parallel to nerves to minimise the risk of this injury. The Cincinnati incision is used for soft tissue release around the hindfoot for paediatric club foot surgery [23]. Although this semi-circumferential incision is almost perpendicular to the course of the sural nerve, at this level the nerve has split into multiple small branches and we note that distal numbness has not been reported by our patients.

**Figure 9:**
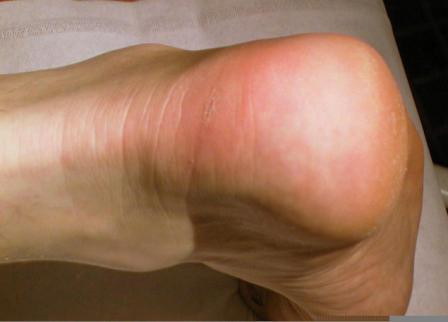
Several months after surgery the mature transverse scar is barely visible.

Transverse scars in the hind foot are difficult to identify once they have matured, and they are also not as prone to the problems of tethering and contracture which may occur with longitudinal scar tissue. Therefore, a transverse semi-circumferential scar may be cosmetically more pleasing, and, once healed, difficult to identify even at close inspection (Figure 9).

All our patients returned to their pre-injury activities, at an average of 9 months following surgery (range 6–15 months). Complications included one case of delayed wound healing with no growth from bacterial swabs and four cases of wound infection. Two of these cultured Staphylococcus Epidermidis and one cultured Enterococcus, all settling with oral antibiotics. A further case cultured Pasteurella. The patient confessed that her cat and dog had been licking her wound which required debridement and eventually the infection settled after 5 months. In no instance was a nerve injury reported.

## Conclusion

The Cincinnati incision allows adequate exposure, has minimal risk of symptomatic iatrogenic nerve injury, and has minimal problems related to the scar.

## Competing interests

The authors declare that they have no competing interests.

## Authors' contributions

MC has performed the literature review and written the technical advance. NM has developed the technique, has reported extensively on Achilles tendon problems, and has collaborated in writing and editing the manuscript. Both the authors have read and approved the final manuscript.

## Pre-publication history

The pre-publication history for this paper can be accessed here:


